# Rheological Restoration and Multi-Criteria Dosage Optimization of Aged SBS-Modified Asphalt Using an Epoxy-Based Reactive Rejuvenator

**DOI:** 10.3390/ma19163543

**Published:** 2026-08-21

**Authors:** Wenwen Jiang, Chunpeng Yan, Jiahao Ji, Ning Li, Jiandong Huang

**Affiliations:** 1 Nanchang East Management Center, Jiangxi Communications Investment Group Co., Ltd., Nanchang 333133, China; 15879089693@163.com; 2 Jiangxi Communications Investment Maintenance Technology Group Co., Ltd., Nanchang 333133, China; 3 Institute of Nuclear Science and Technology, University of South China, Hengyang 421001, China; 4 College of Civil and Transportation Engineering, Hohai University, Nanjing 210098, China; 5 School of Civil Engineering, Guangzhou University, Guangzhou 510006, China

**Keywords:** aged SBS-modified asphalt, epoxy-based reactive rejuvenator, rheological restoration, dosage optimization, entropy-weight TOPSIS, molecular-weight distribution

## Abstract

**Highlights:**

A reactive rejuvenator was designed to restore aged SBS-modified asphalt in high-RAP recycling. The epoxy-based component contributed to the partial recovery of degraded SBS structure. The reactive rejuvenator improved low-temperature relaxation, energy dissipation and fatigue resistance. Entropy-weight TOPSIS identified 6% as the optimum dosage for balanced binder performance. GPC analysis explained the optimum dosage through asphalt-phase adjustment and SBS structure recovery.

The main findings are as follows:

- The reactive rejuvenator restored the viscoelastic properties of aged SBS-modified asphalt.
- The epoxy-based component promoted partial recovery of degraded SBS structure.
- The rejuvenator improved low-temperature, fatigue and energy-dissipation performance.
- A 6% dosage achieved the best performance balance, governed by asphalt-phase adjustment and SBS structure recovery.

The main findings are summarized below:

- The recoverable SBS structure indicates that aged SBS-modified asphalt in high-RAP mixtures can be reused in higher-value pavement applications rather than downgraded use.
- The entropy-weight TOPSIS method provides a practical way to optimize reactive rejuvenator dosage by balancing high-temperature, low-temperature, fatigue and workability performance.

**Abstract:**

High reclaimed asphalt pavement (RAP) contents are often limited by insufficient restoration of field-aged SBS-modified asphalt and the lack of a comprehensive method for rejuvenator dosage selection. This study aimed to develop a multi-performance-based approach for determining the dosage of an epoxy-based reactive rejuvenator under high-RAP conditions. Rejuvenated binders with different dosages were evaluated using conventional tests, DSR, MSCR, BBR, and LAS tests. Continuous low-temperature grading temperature, dissipated energy ratio, and entropy-weight TOPSIS were used for comprehensive evaluation, while GPC was employed to characterize molecular-weight distribution. The rejuvenator improved low-temperature relaxation, fatigue resistance, energy dissipation, and workability, whereas excessive dosages reduced rutting resistance and elastic recovery. Entropy-weight TOPSIS ranked RA-6 highest, with a relative closeness coefficient of 0.66504, and this ranking was consistent with the overall trends obtained from individual performance tests, supporting the feasibility of the proposed evaluation method. GPC results showed systematic changes in molecular-weight distribution after rejuvenation. For the investigated material system, 6% is recommended among the tested dosages. The proposed framework provides a practical basis for dosage determination when material characteristics and performance requirements vary.

## 1. Introduction

With the continuous expansion of pavement maintenance activities, large quantities of reclaimed asphalt pavement (RAP) are generated each year. The high-value utilization of RAP has become an effective strategy for reducing the consumption of virgin aggregates and asphalt, lowering construction costs, and mitigating environmental impacts [1,2,3]. Driven by carbon reduction targets and the growing demand for sustainable transportation infrastructure, conventional recycling practices with low RAP contents are no longer sufficient to fully exploit the resource value of reclaimed materials. Consequently, increasing RAP content has emerged as a major trend in the development of asphalt pavement recycling technologies [4]. However, higher RAP contents inevitably increase the proportion of aged asphalt in recycled mixtures, resulting in increased stiffness, reduced workability, and greater susceptibility to low-temperature and fatigue cracking [5,6,7,8,9]. Therefore, effectively restoring the activity and viscoelastic properties of aged asphalt is critical for the successful engineering application of high-RAP recycled asphalt mixtures.

Conventional rejuvenators are typically composed of light oils, aromatic fractions, or bio-oils, and primarily function by replenishing the light components lost during aging, thereby reducing viscosity and improving low-temperature flexibility [10,11,12,13]. While such rejuvenators can partially restore the rheological properties of aged virgin asphalt, their effectiveness in rejuvenating aged SBS-modified asphalt remains limited. This is because the aging of SBS-modified asphalt involves not only the loss of light fractions and the aggregation of asphaltenes within the asphalt phase, but also the degradation of SBS molecular chains, destruction of the polymer network, and deterioration of phase continuity [14,15,16]. Consequently, the softening and dilution effects provided by conventional rejuvenators alone are often insufficient to simultaneously recover both the asphalt matrix and the SBS-modified structure, and excessive addition may further compromise rutting resistance at high temperatures [17,18,19]. To address these limitations, reactive rejuvenators containing functional groups have attracted increasing attention. Such rejuvenators generally combine light components with reactive species, such as epoxy or isocyanate groups. In addition to restoring the compositional balance and workability of aged asphalt, these reactive groups may interact with functional groups generated during the aging process, facilitating the partial reconstruction of the degraded SBS network. As a result, reactive rejuvenators offer the potential to achieve simultaneous restoration of asphalt properties and repair of the SBS-modified structure [15,20].

Determining the appropriate rejuvenator dosage is critical for achieving satisfactory performance in high-RAP recycled asphalt mixtures. Existing approaches generally rely on empirical recommendations, restoration of conventional properties, rheological performance evaluation, or balanced mix design concepts. The simplest method is to adopt manufacturer-recommended dosages or engineering experience; however, its applicability is often limited by variations in RAP source, aging severity, virgin asphalt characteristics, and rejuvenator composition [6,21]. To improve dosage selection, many studies have determined rejuvenator contents by restoring conventional properties such as penetration, softening point, ductility, and viscosity to target values of virgin asphalt [21,22,23]. Nevertheless, these methods primarily focus on consistency recovery and cannot adequately capture the trade-offs among rutting resistance, cracking resistance, and fatigue performance. More recently, rheology-based approaches have been widely adopted. Performance indicators derived from DSR, BBR, MSCR, and LAS tests, including rutting factor, non-recoverable creep compliance, recovery rate, low-temperature stiffness, m-value, Glover–Rowe parameter, master curves, and PG, have been used to identify suitable dosage ranges [14,19,21,24,25]. In addition, the balanced mix design concept has been introduced to define an acceptable dosage window by simultaneously considering high-temperature deformation resistance and cracking performance [21,26]. Furthermore, chemical and microstructural characterization techniques, such as FTIR, SARA fraction analysis, AFM, GPC, surface free energy, and Hansen solubility parameters, have been employed to evaluate the rejuvenation effectiveness from compositional and structural perspectives [27,28,29,30,31].

Despite considerable progress, several limitations remain in current approaches for determining rejuvenator dosage. Conventional-property-based methods primarily focus on restoring penetration, softening point, ductility, or viscosity, which may lead to satisfactory recovery of individual indices while overlooking the balance among rutting resistance, cracking resistance, and fatigue performance [17,21]. Although rheological and PG-based methods provide a more comprehensive assessment of asphalt performance, dosage selection is still commonly based on achieving predefined target values, making it difficult to reconcile the trade-offs among different performance requirements [24,32]. These limitations become more pronounced for reactive rejuvenators. Unlike conventional rejuvenators, their effectiveness depends not only on the replenishment of light components but also on interactions between reactive functional groups and aged asphalt systems. Consequently, the optimum dosage is governed by both performance restoration and reaction-related effects. Excessive dosage may introduce surplus light fractions or unreacted active components, potentially resulting in adverse effects on performance [15,20]. However, existing evaluation methods rarely consider the relationship between macroscopic performance evolution and microscopic structural changes during dosage determination. As a result, the interpretation of the optimum dosage often remains empirical and lacks sufficient mechanistic support.

**Figure 1:**
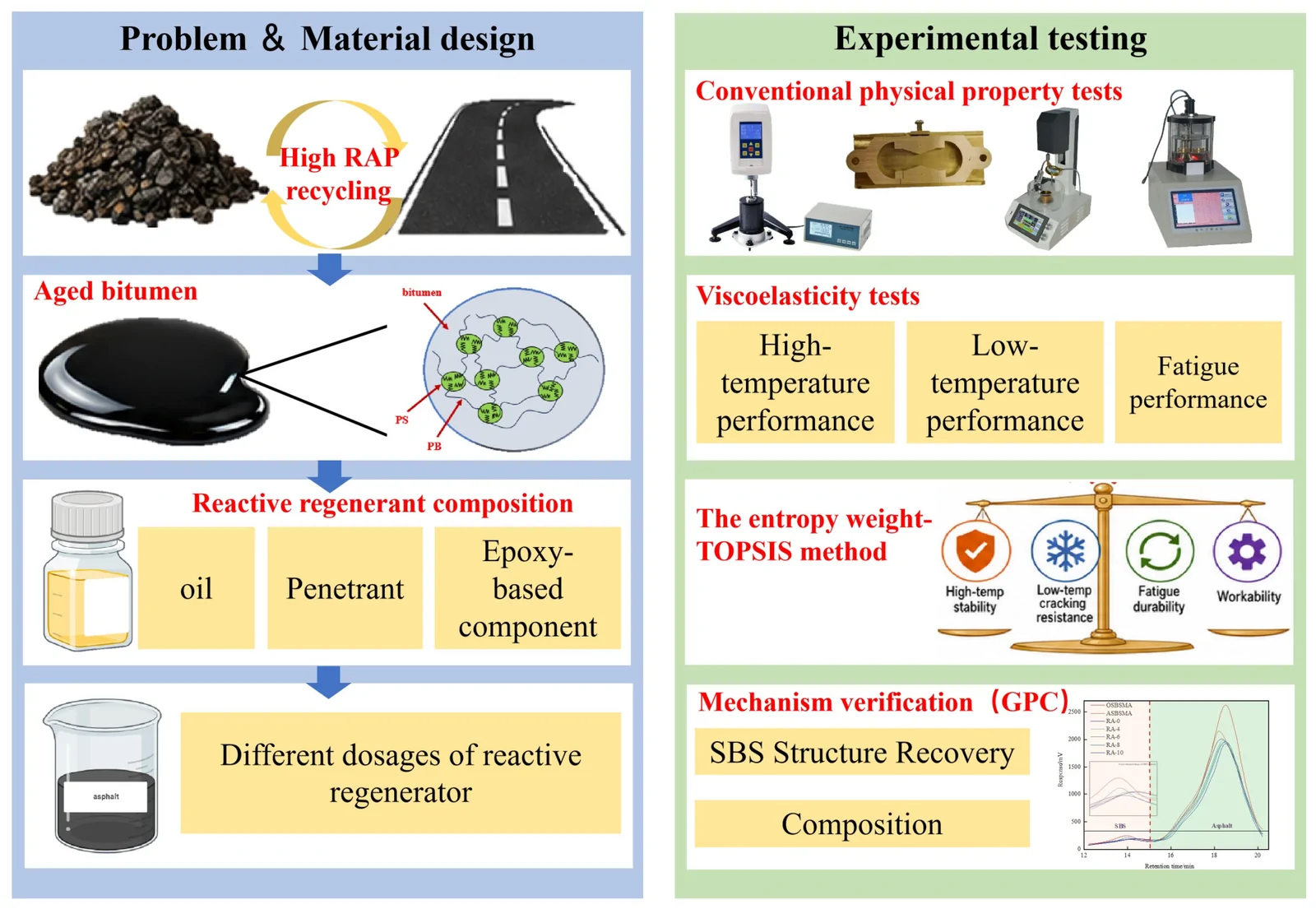
The flowchart for the study.

Despite recent progress, the dosage determination of reactive rejuvenators for aged SBS-modified asphalt still lacks a comprehensive approach that can balance high-temperature, low-temperature, fatigue, and workability requirements. Therefore, this study aims to develop a multi-performance-based dosage evaluation method for an epoxy-based reactive rejuvenator under high-RAP conditions. Conventional, DSR, MSCR, BBR, and LAS tests were conducted to characterize performance changes with rejuvenator dosage, while entropy-weight TOPSIS was used for comprehensive evaluation and GPC for molecular-weight-distribution analysis. The consistency between the TOPSIS ranking and the individual performance trends supports the feasibility of the proposed method for rejuvenator dosage determination. The flowchart for this study is presented in Figure 1.

## 2. Materials and Methods

### 2.1. Materials

#### 2.1.1. Asphalt

The asphalt used in this study included virgin SBS-modified asphalt and aged SBS-modified asphalt (Jiangsu Suzhou High-tech Materials Co., Ltd, Suzhou, China). The recovered SBS-modified asphalt was obtained from an SMA-13 asphalt pavement on the Runyang Yangtze River Bridge in Jiangsu Province, which had been in service for 11 years and is located in climate zone 1-3-2. Experimental results indicated that the properties of the aged asphalt, including penetration, softening point, ductility, and viscosity, had significantly changed. Notably, the penetration and ductility no longer met the current requirements for asphalt binder standards. The properties of the virgin asphalt and aged asphalt are listed in Table 1.

#### 2.1.2. Reactive Rejuvenator

An epoxy-based reactive rejuvenator was formulated using waste engine oil, a penetrant, and an epoxy-containing material. Waste engine oil was first heated to 120 °C, followed by the addition of the epoxy-containing material and mixing at 500 rpm for 15 min. The mixture was then cooled to 70 °C, after which the penetrant was incorporated and blended at 500 rpm for another 15 min. The resulting product was used as the reactive rejuvenator in this study. Its basic properties are presented in Table 2.

#### 2.1.3. Sample Preparation

Original SBS-modified asphalt (OSBSMA) and aged SBS-modified asphalt (ASBSMA) were heated to 175 °C until a fluid state was achieved. The two asphalts were then blended at a mass ratio of OSBSMA = 1:4 and mechanically stirred at 500 rpm for 30 min at 175 °C. The resulting asphalt sample was designated as RA-0. Subsequently, the epoxy-based reactive rejuvenator was added to RA-0 at dosages of 4%, 6%, 8%, and 10% by mass of ASBSMA. The mixtures were mechanically stirred at 500 rpm for 30 min to obtain the rejuvenated asphalt samples, which were designated as RA-4, RA-6, RA-8, and RA-10, respectively.

#### 2.1.4. Research Program

To provide a clear overview of the experimental design, the compositions of the different asphalt samples, the number of specimens, and the corresponding test methods are summarized in Table 3.

### 2.2. Conventional Physical Property Tests

All the samples were conducted physical properties test, including softening point, penetration, ductility and viscosity tests according to the ASTM Standard D36, 2006 [33], ASTM Standard D5, 2013 [34], ASTM Standard D113, 2017 [35], and ASTM Standard D4402, 2023 [36], respectively.

### 2.3. High-Temperature Performance

#### 2.3.1. Temperature Sweep Test

The rheological properties of the asphalt samples were characterized using a dynamic shear rheometer (DSR, TA Instruments, New Castle, DE, USA). Approximately 0.1 g of asphalt sample was placed at the center of the lower parallel plate. After being heated to a fluid state, the upper plate was lowered to the testing position, and any excess material was trimmed. A parallel-plate geometry with a diameter of 25 mm and a gap of 1 mm was employed. Temperature sweep tests were conducted over a temperature range of 58–94 °C at a heating rate of 2 °C/min and an angular frequency of 10 rad/s. In addition, the viscous flow characteristics of the samples were evaluated under steady shear mode.

#### 2.3.2. MSCR Test

The multiple stress creep recovery (MSCR) test was conducted to evaluate the high-temperature viscoelastic response of the asphalt binders. The test was performed using a dynamic shear rheometer (DSR) in creep-recovery mode in accordance with ASTM D7405-15 [37]. Test temperatures of 60 °C and 70 °C were selected. Two stress levels, 0.1 kPa and 3.2 kPa, were applied, with 10 loading cycles conducted at each stress level. Each cycle consisted of a 1 s creep-loading period followed by a 9 s recovery period. Based on the strain response, the average percent recovery (*R*) and non-recoverable creep compliance (*J_nr_*) were calculated to characterize the elastic recovery capability and resistance to permanent deformation of the asphalt binders at high temperatures. The calculation procedures are presented in Equations (1)–(4).

(1) $$J_{nr}(\sigma ,N)=\frac{\varepsilon_{r}-\varepsilon_{0}}{\sigma }$$

(2) $$R(\sigma ,N)=\frac{\varepsilon_{c}-\varepsilon_{r}}{\varepsilon_{c}-\varepsilon_{0}}\times 100$$

(3) $$J_{nr}=\frac{\sum_{N=1}^{10}J_{nr}(\sigma ,N)}{10}$$

(4) $$R=\frac{\sum_{N=1}^{10}R(\sigma ,N)}{10}$$

where *σ* denotes the applied stress level, set at 0.1 kPa or 3.2 kPa; *ε*_0_ denotes the initial strain at the beginning of each cycle (%); *ε_c_* denotes the strain at the end of the creep phase (%); *ε_r_* denotes the strain at the end of the recovery phase (%).

### 2.4. Low-Temperature Performance

#### 2.4.1. BBR Test

A bending beam rheometer (BBR) was employed to evaluate the low-temperature viscoelastic properties of the asphalt binders. The test provided the creep stiffness (*S* value) and creep rate (*m* value), where *S* characterizes the resistance of the binder to deformation under low-temperature loading, and the m value reflects its stress relaxation capability. In general, lower *S* values and higher m values indicate superior low-temperature performance.

The total loading duration was 240 s, including a 5 s loading ramp period followed by a constant load of 980 mN. According to the Strategic Highway Research Program (SHRP) specifications [38], an asphalt binder is considered to satisfy the low-temperature performance requirement when the creep stiffness at 60 s is no greater than 300 MPa and the m value is no less than 0.30.

#### 2.4.2. Continuous Low-Temperature Grading Temperature (T_C_)

In the SHRP performance grading (PG) system, low-temperature grades are assigned at 6 °C intervals. Consequently, asphalt binders classified within the same PG low-temperature grade may still exhibit different levels of low-temperature performance. To provide a more sensitive assessment of low-temperature behavior, the continuous low-temperature grading temperature (*T_c_*) was calculated based on the BBR test results. The calculation procedure is expressed in Equations (5)–(7).

(5) $$T_{C}(S)=T_{1}+\left[\frac{\mathrm{lg}(300)-\mathrm{lg}(S_{1})}{\mathrm{lg}(S_{1})-\mathrm{lg}(S_{2})}\times (T_{2}-T_{1})\right]-10$$

(6) $$T_{C}(m)=T_{1}+\left[\frac{0.3-m_{1}}{m_{1}-m_{2}}\times (T_{2}-T_{1})\right]-10$$

(7) $$T_{C}=\max\left[T_{C}(S),T_{C}(m)\right]$$

where *T_C_*(*S*) is the continuous grading temperature corresponding to a *S* value of 300 Mpa (°C); *T_C_*(*m*) is the continuous grading temperature corresponding to a *m* value of 0.30 (°C); *T*_1_ is the test temperature that satisfies the SHRP low-temperature grading requirement (°C); *T*_2_ is equal to (*T*_1_-_6_) (°C); *S*_1_ and *S*_2_ are the creep stiffness values measured at *T*_1_ and *T*_2_, respectively (MPa); and *m*_1_ and *m*_2_ are the corresponding m values at *T*_1_ and *T*_2_, respectively.

The continuous low-temperature grading temperature *T_C_* is determined as the higher value of *T_C_*(*S*) and *T_C_*(*m*).

#### 2.4.3. Dissipated Energy Ratio (DER)

The Burgers model, which consists of a Maxwell element and a Kelvin element connected in series, is widely used to characterize the low-temperature creep behavior of asphalt binders. It can effectively describe the instantaneous elastic deformation, delayed elastic deformation, and viscous flow deformation that occur during the creep process. Therefore, in this study, the Burgers model was employed to fit the creep compliance curves obtained from the BBR tests. The mathematical expression of the model is given in Equation (8).

(8) $$J\left(t\right)=\frac{1}{S(t)}=\frac{\varepsilon (t)}{\sigma }=\frac{1}{E_{1}}+\frac{t}{\eta_{1}}+\frac{1}{E_{2}}(1-e^{-\frac{E_{2}}{\eta_{2}}t})$$

where *t* is the creep time (s); *J*(*t*) is the creep compliance of the asphalt binder (1/MPa); *S*(*t*) is the creep stiffness (MPa); *ε*(*t*) is the creep strain; and *E*_1_, *E*_2_, *η*_1_, and *η*_2_ are the elastic and viscous parameters of the Burgers model.

From the perspective of energy conversion, the energy induced in an asphalt binder under external loading can be divided into stored energy and dissipated energy. The stored energy is retained within the material in the form of elastic strain energy, whereas the dissipated energy is associated with viscous flow and internal structural rearrangement. At low temperatures, the cracking resistance of asphalt binders depends not only on their deformation capacity but also on their ability to dissipate input energy and alleviate stress concentration. Therefore, DER is defined as the ratio of dissipated energy to stored energy, and was introduced to characterize the energy dissipation capability of asphalt binders during the low-temperature creep process. Based on the fitting parameters obtained from the Burgers model, the expressions for stored energy, dissipated energy, and DER are given in Equations (9)–(11), respectively.

(9) $$W_{s}(t)=\sigma^{2}\left[\frac{1}{E_{1}}+\frac{1}{2E_{2}}{\left(1-e^{-\frac{E_{2}}{\eta_{2}}t}\right)}^{2}\right]$$

(10) $$W_{d}(t)=\sigma^{2}\left[\frac{t}{\eta_{1}}+\frac{1}{2E_{2}}(1-e^{-2\frac{E_{2}}{\eta_{2}}t})\right]$$

(11) $$DER=\frac{W_{d}(t)}{W_{s}(t)}=\frac{\left[\frac{t}{\eta_{1}}+\frac{1}{2E_{2}}(1-e^{-2\frac{E_{2}}{\eta_{2}}t})\right]}{\left[\frac{1}{E_{1}}+\frac{1}{2E_{2}}{\left(1-e^{-\frac{E_{2}}{\eta_{2}}t}\right)}^{2}\right]}$$

where *W_s_*(*t*) and *W_d_*(*t*) represent the stored energy and dissipated energy, respectively, and DER denotes the dissipated energy ratio. A higher DER indicates a greater capacity of the asphalt binder to dissipate externally applied energy during creep loading, which contributes to stress relaxation and improves resistance to low-temperature cracking.

### 2.5. Fatigue Performance

The linear amplitude sweep (LAS) test was conducted to evaluate the fatigue performance of the asphalt binders [39]. The test was performed under strain-controlled loading and consisted of two stages: a frequency sweep and an amplitude sweep. First, a frequency sweep was carried out at a strain level of 0.1% over a frequency range of 0.2–30 Hz to determine the linear viscoelastic properties of the material. Subsequently, an amplitude sweep was performed at a constant frequency of 10 Hz, during which the strain amplitude increased linearly from 0.1% to 30% over a period of 300 s, allowing the damage evolution behavior of the binder under cyclic loading to be characterized.

Based on the Viscoelastic Continuum Damage (VECD) theory, the fatigue damage process was described using the integrity parameter and the accumulated damage parameter. The fatigue life *N_f_* was then calculated in accordance with the relevant specifications [39]. In this study, the fatigue lives at strain levels of 2.5% and 5% were selected as the primary indicators for evaluating fatigue performance.

### 2.6. Multi-Criteria Evaluation Using the Entropy-Weight TOPSIS Method

The entropy-weight TOPSIS method was employed to comprehensively evaluate the performance of rejuvenated asphalt samples and determine the optimal rejuvenator dosage. This approach combines the objective weighting capability of the entropy-weight method with the ranking advantages of TOPSIS, thereby reducing subjective bias in the evaluation process. Assuming that *m* alternatives are evaluated using *n* performance indicators, the initial decision matrix can be expressed as *X* = (*x_ij_*)*_n_*_×*m*_*,* where *x_ij_* denotes the value of the jth indicator for the ith alternative. All indicators included in the initial TOPSIS decision matrix were based on valid experimental or calculated results, and no missing values were present. Therefore, no missing-data imputation or substitution was required in the TOPSIS analysis.

First, the decision matrix was normalized to eliminate the influence of different dimensions among evaluation indicators. The normalization procedures for benefit-type and cost-type indicators are given in Equations (12) and (13), respectively.

(12) $$b_{ij}=\frac{x_{ij}-\min_{j}(x_{ij})}{\max_{j}(x_{ij})-\min_{j}(x_{ij})}$$

(13) $$b_{ij}=\frac{\max_{j}(x_{ij})-x_{ij}}{\max_{j}(x_{ij})-\min_{j}(x_{ij})}$$

where max*_j_*(*x_ij_*) and min*_j_*(*x_ij_*) denote the maximum and minimum values of the jth indicator, respectively.

Subsequently, the entropy value, degree of variation, and entropy weight of each indicator were calculated according to Equations (14)–(16). The entropy-weight method assigns larger weights to indicators with greater discrimination ability among alternatives.

(14) $$y_{ij}=\frac{b_{ij}}{\sum_{i=1}^{n}b_{ij}}$$

(15) $$H_{j}=-\frac{1}{\ln n}\sum_{i=1}^{n}y_{ij}\ln y_{ij}$$

(16) $$\omega_{j}=\frac{1-H_{j}}{\sum_{i=1}^{m}\left(1-H_{j}\right)}$$

where *y_ij_* is the proportion of the jth indicator for the ith alternative, *H_j_* is the entropy value of the jth indicator, and *ω_j_* is the corresponding entropy weight.

Based on the obtained weights, the weighted normalized decision matrix was constructed according to Equation (17).

(17) $$z_{ij}=\omega_{j}\cdot b_{ij}$$

The positive ideal solution and negative ideal solution were then determined using Equations (18) and (19), respectively.

(18) $$Z^{+}=(z_{1}^{+},z_{2}^{+},\ldots ,z_{m}^{+})$$

(19) $$Z^{-}=(z_{1}^{-},z_{2}^{-},\ldots ,z_{m}^{-})$$

Thereafter, the Euclidean distances of each alternative from the positive and negative ideal solutions were calculated according to Equations (20) and (21), respectively. A smaller distance from the positive ideal solution and a larger distance from the negative ideal solution indicate better overall performance.

(20) $$D_{i}^{+}=\sqrt{\sum_{j=1}^{m}{\left(z_{ij}-z_{j}^{+}\right)}^{2}}$$

(21) $$D_{i}^{-}=\sqrt{\sum_{j=1}^{m}{\left(z_{ij}-z_{j}^{-}\right)}^{2}}$$

Finally, the relative closeness coefficient was calculated using Equation (22). A larger closeness coefficient indicates that the alternative is closer to the positive ideal solution and exhibits superior comprehensive performance.

(22) $$C_{i}=\frac{D_{i}^{-}}{D_{i}^{+}+D_{i}^{-}}$$

### 2.7. Gel Permeation Chromatography

Gel permeation chromatography (GPC) was employed to characterize the molecular-weight distribution of the rejuvenated asphalt binders [40]. The molecular-weight distribution can provide information on molecular-structure changes associated with the rejuvenation process and thereby assist in interpreting the corresponding rheological performance restoration. Therefore, the variations in different molecular-weight fractions with rejuvenator dosage were analyzed in this study. Binder samples were dissolved in 15 mL of tetrahydrofuran (THF) at a concentration of 2–3 wt.% and stored for 3 days to ensure complete dissolution of both the asphalt components and SBS modifier. Prior to testing, the solution was filtered through a 0.45 μm membrane filter (GPC, Agilent 1260 Infinity II, Santa Clara, CA, USA)to remove insoluble impurities. Subsequently, 0.5 mL of the filtered solution was injected into the GPC system for analysis. The measurements were conducted at 25 °C with a mobile-phase flow rate of 1.0 mL/min.

## 3. Results and Discussion

### 3.1. Conventional Physical Properties

**Figure 2:**
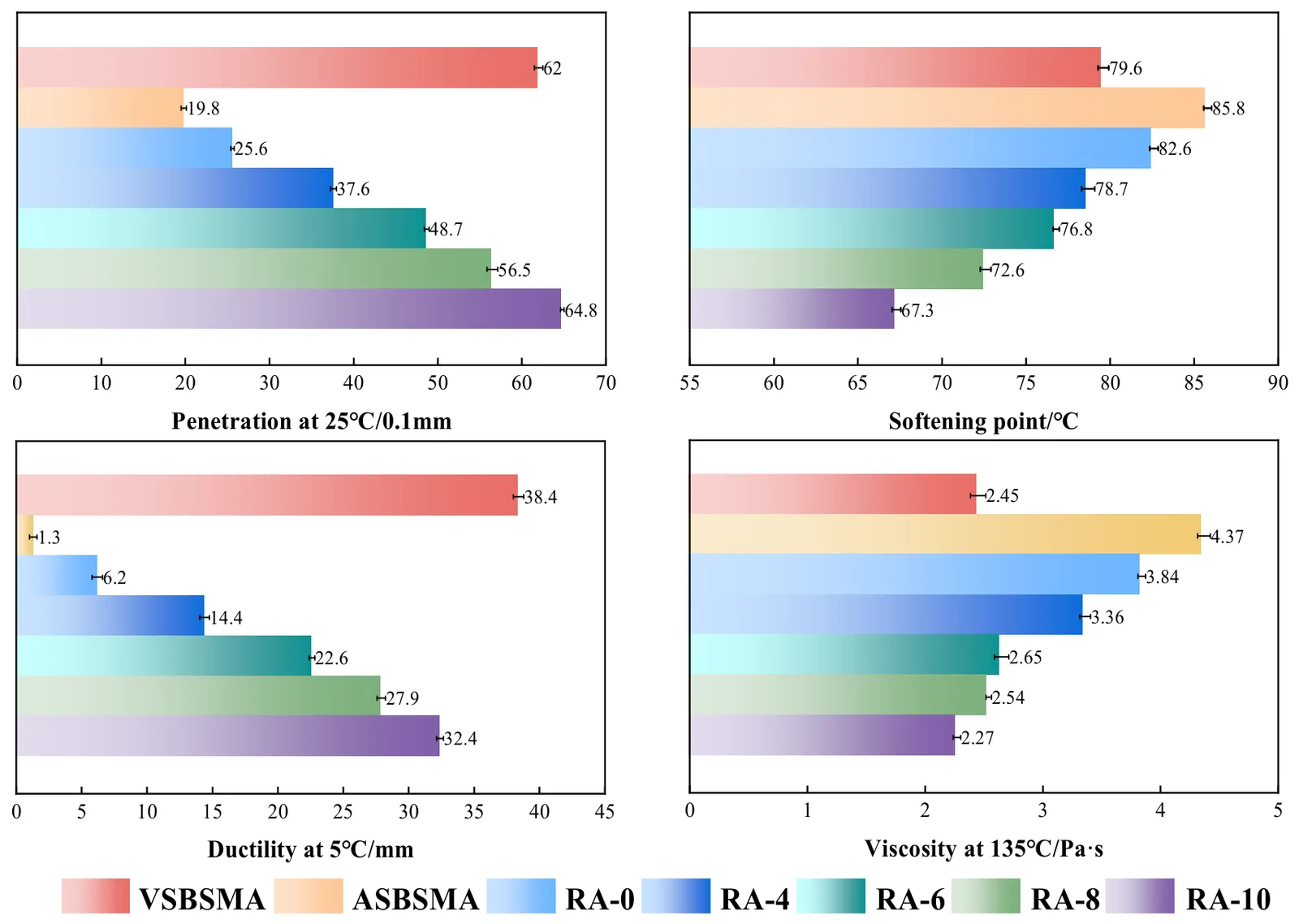
Conventional physical properties of different asphalt samples.

Figure 2 presents the penetration at 25 °C, softening point, ductility at 5 °C, and rotational viscosity at 135 °C of the different asphalt samples. ASBSMA exhibited the highest softening point and rotational viscosity, but the lowest penetration and ductility, indicating that field aging significantly increased asphalt stiffness and enhanced high-temperature stability while markedly deteriorating low-temperature flexibility and workability. Compared with ASBSMA, RA-0 showed a substantial increase in ductility from 1.3 cm to 6.2 cm, suggesting that the incorporation of virgin SBS-modified asphalt could partially restore the flexibility of the aged asphalt; however, the recovery remained insufficient to achieve the ductility level of the original asphalt. As the dosage of the reactive rejuvenator increased, penetration and ductility gradually increased, whereas softening point and rotational viscosity decreased. These results indicate that the rejuvenator effectively improved low-temperature flexibility and construction workability, but at the expense of high-temperature stability. This behavior can be attributed to the replenishment of light fractions by the waste engine oil component, which promotes the dispersion of asphaltene aggregates and reduces asphalt stiffness. Meanwhile, the epoxy-containing component may interact with aged SBS chains and polar constituents in the asphalt system, facilitating partial restoration of the modified structure. Consequently, the conventional properties of the rejuvenated asphalt were significantly improved. It is noteworthy that the improvements in low-temperature flexibility and workability were accompanied by a reduction in high-temperature stability, highlighting a clear performance trade-off associated with rejuvenator addition. Therefore, an optimum dosage is required to achieve a balance among rutting resistance, low-temperature cracking resistance, and workability.

### 3.2. High-Temperature Performance

#### 3.2.1. Rutting Factor and Failure Temperature

The rutting factor (G*/sinδ) is widely used to characterize the resistance of asphalt binders to permanent deformation at elevated temperatures, with higher values indicating superior rutting resistance. In this study, the temperature corresponding to G*/sinδ = 1 kPa was defined as the failure temperature.

**Figure 3:**
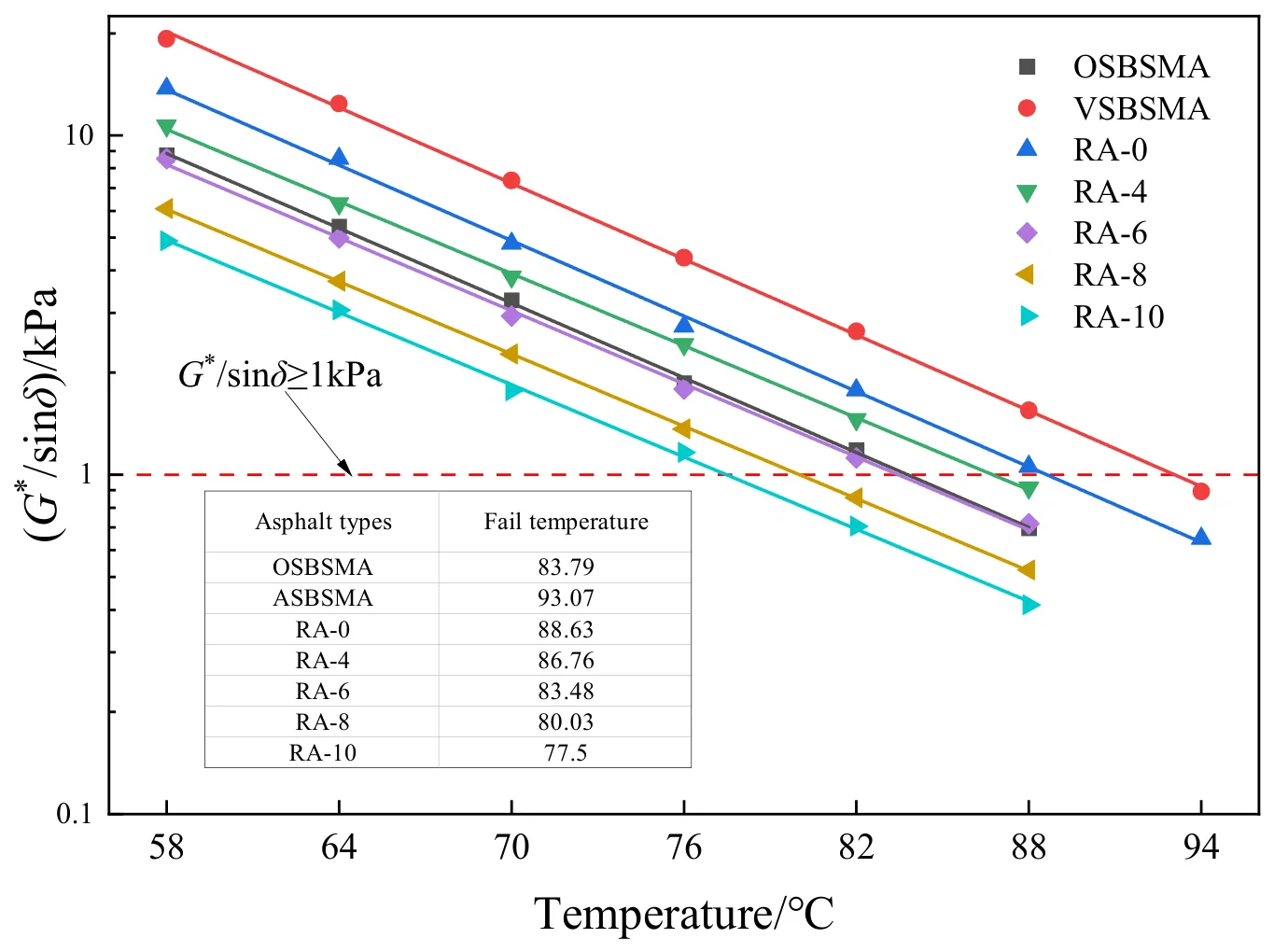
Rutting factor and failure temperature of different asphalt samples.

As shown in Figure 3, ASBSMA exhibited the highest G*/sinδ values throughout the tested temperature range, with a failure temperature of 93.07 °C. This result indicates that field aging substantially increased the stiffness and deformation resistance of the SBS-modified asphalt, which is consistent with the softening point results discussed previously. In contrast, the addition of the reactive rejuvenator progressively reduced both the rutting factor and failure temperature. The failure temperature decreased from 88.63 °C for RA-0 to 77.50 °C for RA-10, representing a reduction of 15.57 °C relative to ASBSMA. The observed decline in high-temperature performance can be primarily attributed to the softening effect of the rejuvenator. The waste engine oil component replenishes lost light fractions and promotes the dispersion of asphaltene-rich structures, thereby reducing asphalt stiffness. Nevertheless, the reduction in rutting resistance was not proportional to the rejuvenator dosage. Compared with the other rejuvenated asphalt, RA-6 exhibited a G*/sinδ-temperature relationship and failure temperature that were closest to those of OSBSMA, suggesting that a dosage of 6% provided the most effective restoration of the original high-temperature rheological characteristics. This behavior reflects the competing effects of the reactive rejuvenator. While the light components enhance fluidity and reduce stiffness, the epoxy functional component may facilitate interactions with aged SBS fragments and polar species in the asphalt, contributing to partial reconstruction of the modified network structure. Consequently, an appropriate rejuvenator dosage can improve workability while preserving adequate high-temperature stability, whereas excessive addition causes the softening effect to dominate, resulting in a pronounced deterioration of rutting resistance.

#### 3.2.2. *J_nr_* and *R*

**Figure 4:**
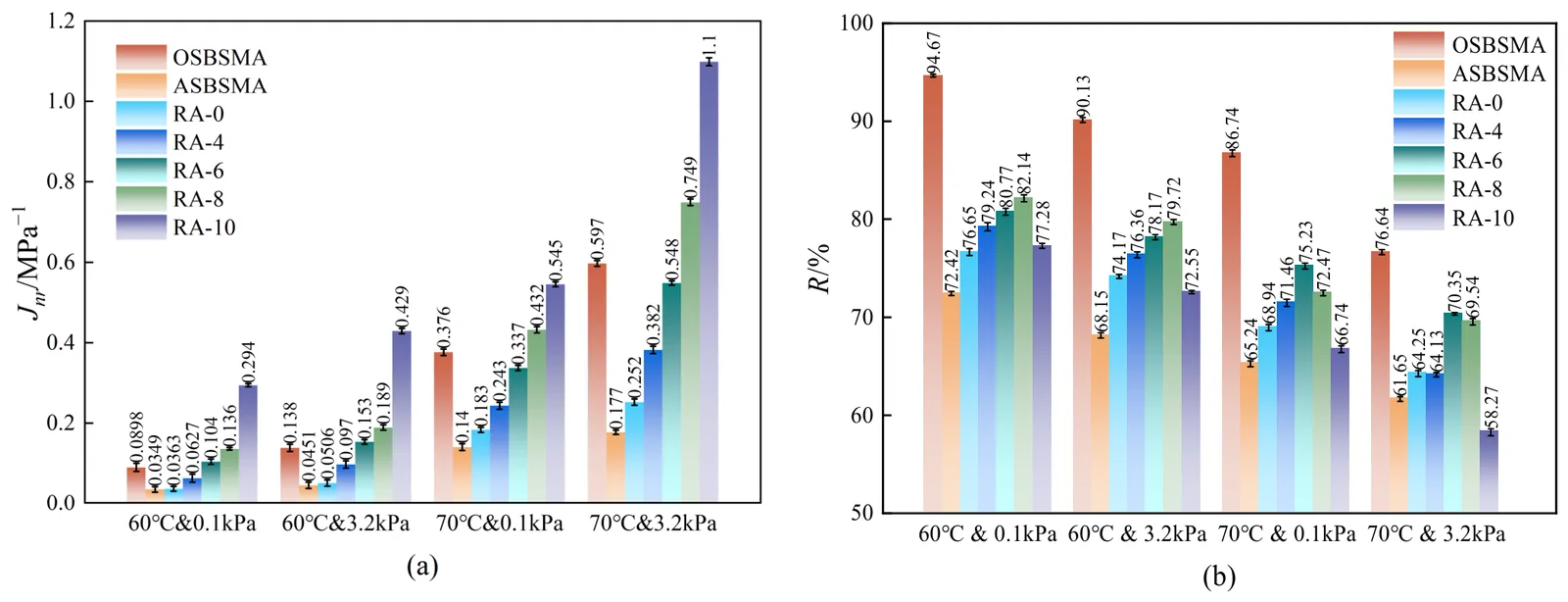
*J_nr_* (**a**) and *R* (**b**) of different asphalt samples.

Figure 4 presents the *J_nr_* and *R* of the different asphalt samples measured at 60 °C and 70 °C under stress levels of 0.1 kPa and 3.2 kPa. The parameter *J_nr_* characterizes the susceptibility of asphalt to permanent deformation, with lower values indicating better rutting resistance. In contrast, R reflects the elastic recovery capability of asphalt, and higher values correspond to greater recoverable deformation.

As shown in Figure 4, ASBSMA exhibited relatively low *J_nr_* and *R* values. The low *J_nr_* values indicate that field aging substantially increased asphalt stiffness and enhanced resistance to permanent deformation at high temperatures. However, the reduced *R* values suggest that aging simultaneously weakened the elastic response of the SBS-modified asphalt. Compared with ASBSMA, RA-0 showed a pronounced increase in *R*, indicating that the incorporation of virgin SBS-modified asphalt partially restored the elastic recovery capability of the aged asphalt. With increasing rejuvenator dosage, *J_nr_* generally increased, whereas *R* exhibited an initial increase followed by a decline. These results indicate that the reactive rejuvenator improved viscoelastic recovery while progressively reducing resistance to permanent deformation. The highest *R* values were observed at approximately 8% dosage under 60 °C and 6% dosage under 70 °C. However, when the dosage increased to 10%, a noticeable reduction in *R* occurred, suggesting deterioration of elastic recovery due to excessive rejuvenator addition. The observed behavior can be attributed to the competing effects of the reactive rejuvenator. The epoxy functional component may promote interactions among aged SBS fragments and polar species, thereby enhancing elastic recovery. In contrast, excessive light fractions intensify the softening effect and increase irreversible deformation under loading. Consequently, an appropriate rejuvenator dosage is required to achieve a balance between permanent deformation resistance and elastic recovery at high temperatures.

### 3.3. Low-Temperature Performance

#### 3.3.1. *S* Value, *m* Value and *T_c_*

**Figure 5:**
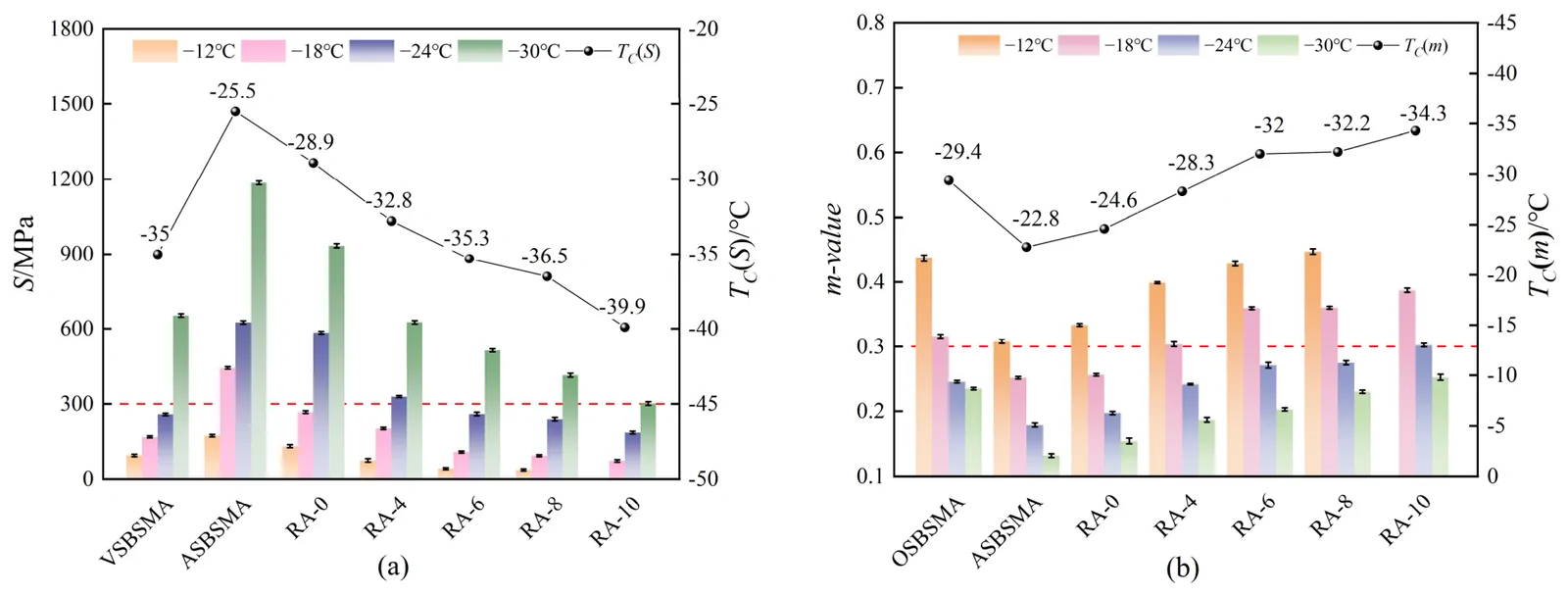
*S* values (**a**) and *m* values (**b**) of different asphalt samples.

The BBR results are presented in Figure 5. ASBSMA exhibited the highest *S* value and the lowest *m* value at all test temperatures, indicating that field aging significantly increased the low-temperature stiffness of the asphalt while reducing its stress relaxation capability. Compared with ASBSMA, RA-0 showed lower *S* values and higher *m* values, suggesting that the incorporation of virgin SBS-modified asphalt could partially improve the low-temperature performance of the aged asphalt, although the extent of recovery remained limited. As the dosage of the reactive rejuvenator increased, the *S* value continuously decreased, whereas the *m* value generally increased. These results indicate that the rejuvenator effectively enhanced the low-temperature deformation and stress relaxation capabilities of the asphalt. It should be noted that the deflection of RA-10 at −12 °C reached 5 mm before the end of the 240 s loading period; therefore, no valid BBR result was obtained at this temperature. This invalid data point was not used as an individual indicator in the TOPSIS analysis and was not replaced or imputed. According to the SHRP specification, an asphalt sample should satisfy a creep stiffness not exceeding 300 MPa and *m* value not less than 0.30. Although differences in *S* and *m* values were observed among OSBSMA, RA-4, RA-6, and RA-8, all four samples were classified as PG −28, indicating that the conventional PG system has limited capability to distinguish subtle differences in low-temperature performance. In contrast, *T_c_* provides a more sensitive assessment. ASBSMA exhibited the highest *T_c_*, indicating the poorest low-temperature performance. The *T_c_* of RA-0 decreased by only 1.8 °C relative to ASBSMA, suggesting limited improvement. With increasing rejuvenator dosage, *T_c_* progressively decreased, reaching −34.3 °C for RA-10, which was 11.5 °C lower than that of ASBSMA. This result demonstrates a substantial enhancement in low-temperature cracking resistance.

The observed improvement can be attributed to both compositional adjustment and structural restoration induced by the reactive rejuvenator. The waste engine oil component replenishes the light fractions lost during aging and promotes the dispersion of asphaltene-rich structures, thereby reducing low-temperature stiffness. Meanwhile, the epoxy functional component may interact with aged SBS fragments and polar functional groups, facilitating partial restoration of the modified structure and improving viscoelastic relaxation capability. For all samples, *T_c_*_(_*_m_*_)_ was higher than *T_c_*_(_*_S_*_)_, indicating that the low-temperature performance was primarily governed by the m value. This finding suggests that enhancing stress relaxation capability is the key factor in restoring the low-temperature performance of rejuvenated asphalt.

#### 3.3.2. Creep Compliance and DER

**Figure 6:**
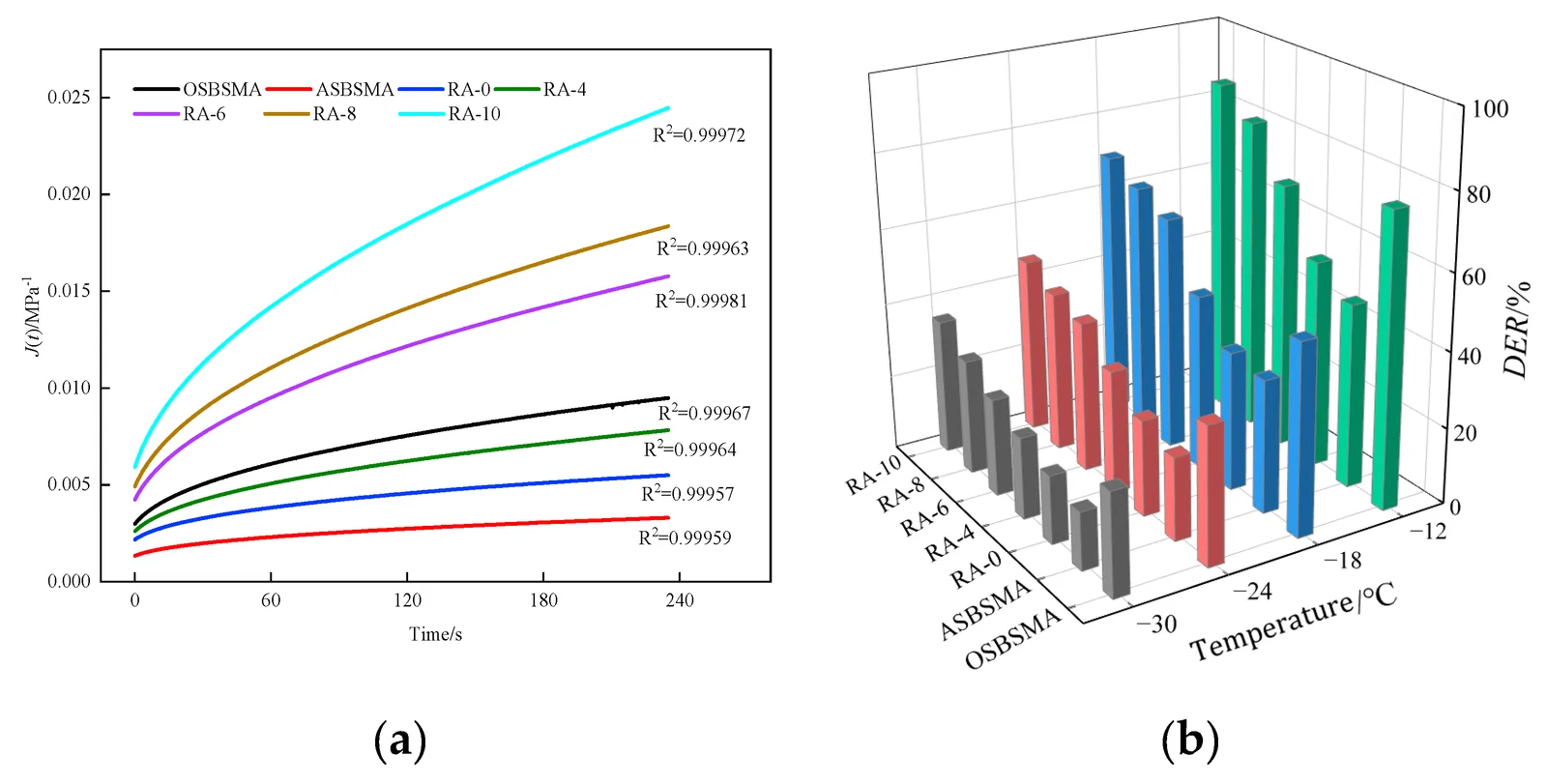
Creep compliance (**a**) and *DER* (**b**) of different asphalt samples.

Figure 6 presents the creep compliance curves at −18 °C and DER at different temperatures for the asphalt samples. ASBSMA exhibited the lowest creep compliance throughout the loading period, indicating that field aging substantially restricted the low-temperature deformability of the asphalt. With increasing dosage of the reactive rejuvenator, the creep compliance gradually increased, suggesting enhanced creep deformation and stress relaxation capabilities at low temperatures. DER represents the ratio of dissipated energy to stored energy during the creep process. For all samples, DER decreased with decreasing temperature, indicating that viscous flow became increasingly restricted at lower temperatures and a greater proportion of the input energy was stored in the form of elastic strain energy. Compared with OSBSMA, ASBSMA exhibited lower DER values, demonstrating that aging reduced the energy dissipation capability of the asphalt. In contrast, DER increased progressively with rejuvenator dosage. At −18 °C, the DER value increased from 36.7% for RA-0 to 70.1% for RA-10, indicating a substantial enhancement in energy dissipation capacity.

The improvement in DER can be attributed to the combined effects of compositional adjustment and structural restoration induced by the reactive rejuvenator. The replenishment of light fractions promotes molecular mobility and facilitates viscous dissipation, while the epoxy functional component may contribute to partial reconstruction of the aged SBS-modified structure. As a result, a larger proportion of the externally applied energy is dissipated rather than stored elastically. Consequently, the rejuvenated asphalt exhibits enhanced stress relaxation capability, reduced stress concentration, and a lower susceptibility to crack initiation under low-temperature conditions.

### 3.4. Fatigue Performance

#### 3.4.1. Stress–Strain Response

**Figure 7:**
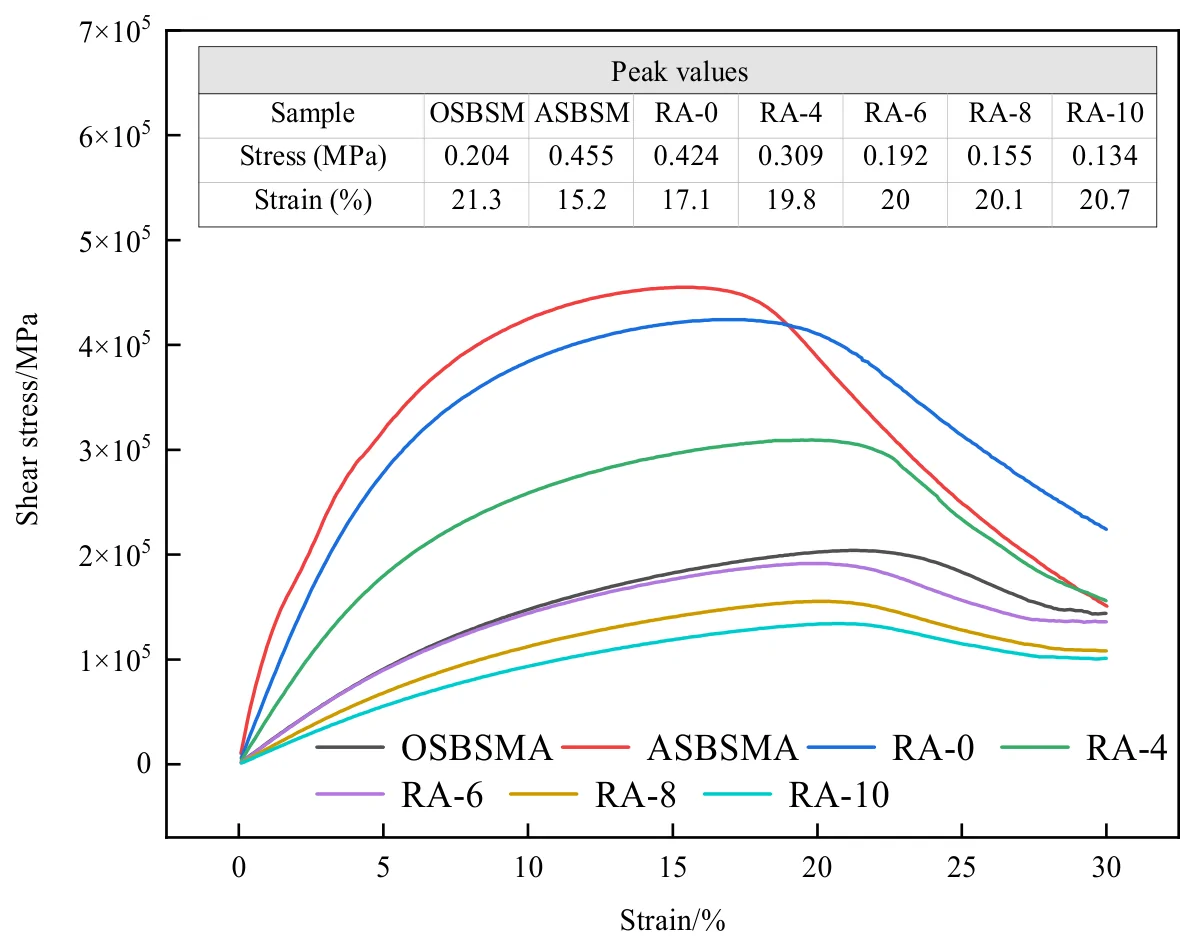
Stress–strain response of different asphalt samples.

Figure 7 presents the shear stress–strain curves of the different asphalt samples. The yield stress of OSBSMA and ASBSMA was 0.204 MPa and 0.455 MPa, respectively, while the corresponding yield strains were 21.3% and 15.2%. These results indicate that aging increased the deformation resistance of the SBS-modified asphalt but significantly reduced its strain tolerance. The yield stress and yield strain of RA-0 were 0.424 MPa and 17.1%, respectively, suggesting that the incorporation of virgin SBS-modified asphalt alone provided only limited improvement in the ductile deformation capacity of the aged asphalt. With increasing dosage of the reactive rejuvenator, the peak stress gradually decreased and the peak position shifted toward higher strain levels. This behavior indicates that the rejuvenator mitigated the brittle and stiff characteristics induced by aging and enhanced the deformation compatibility of the asphalt. When the rejuvenator dosage reached 6% or higher, the stress–strain curves progressively approached that of OSBSMA. Moreover, the post-peak stress decay became more gradual, suggesting an improved ability to release and redistribute stress under large deformations. Such behavior is beneficial for delaying localized damage accumulation and enhancing resistance to fatigue cracking.

The observed changes may be attributed to the combined effects of compositional adjustment and structural restoration induced by the reactive rejuvenator. Aging causes oxidation and chain scission of the polybutadiene (PB) soft segments in SBS, thereby reducing the continuity of the modified network structure. The epoxy functional component may interact with polar functional groups generated during aging, facilitating partial restoration of the SBS-modified structure. Consequently, stress transfer and deformation coordination under large strains are improved, leading to slower post-peak stress decay and enhanced fatigue resistance.

#### 3.4.2. C–D Relationship and Fatigue Life

**Figure 8:**
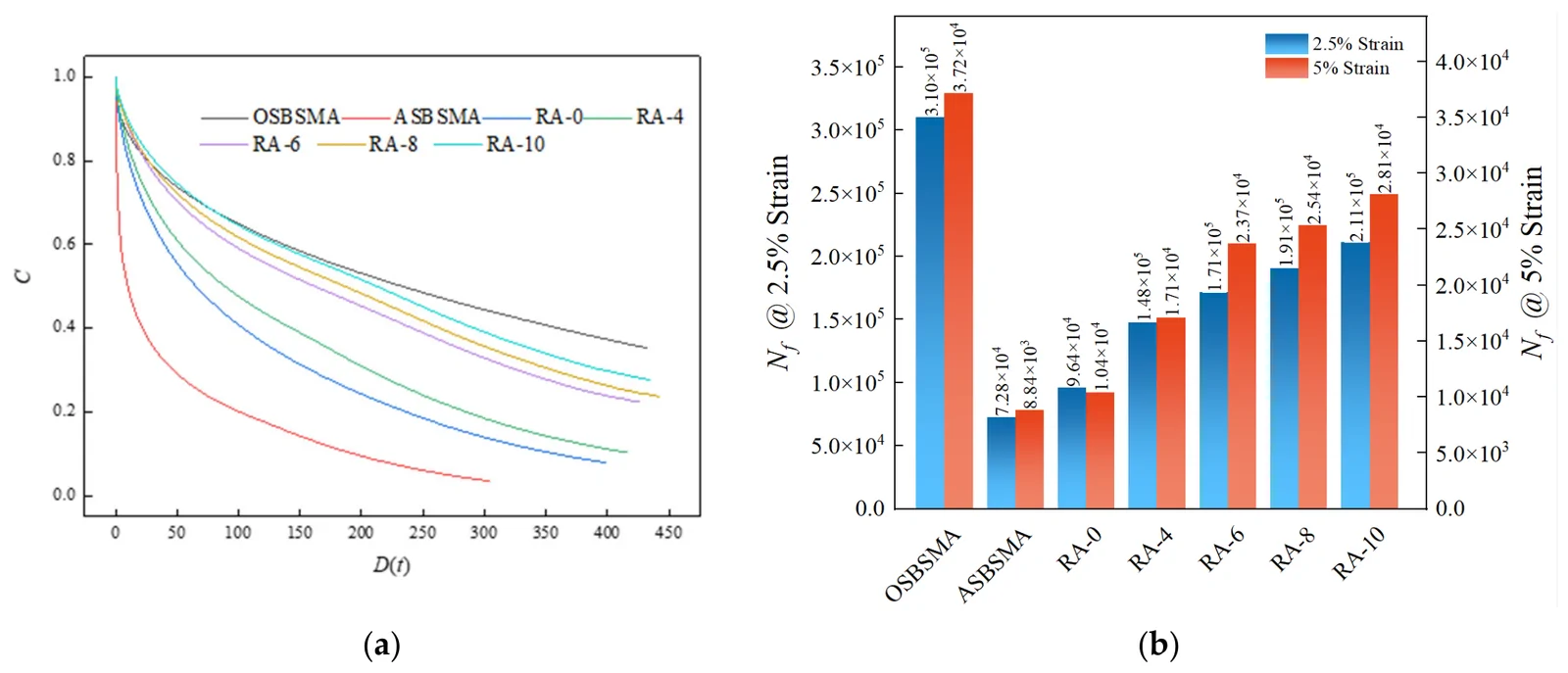
C–D relationship (**a**) and *N_f_* (**b**) of different asphalt samples.

Figure 8 presents the fatigue damage characteristic curves derived from the VECD theory and the *N_f_* at different strain levels. In the C–D curves, the integrity parameter (C) decreased with increasing accumulated damage parameter (D). A steeper decline in C indicates a faster rate of damage accumulation during cyclic loading. ASBSMA exhibited a rapid reduction in C at the early stage of loading, indicating a higher susceptibility to fatigue damage after field aging. In contrast, the incorporation of virgin asphalt and the reactive rejuvenator significantly reduced the degradation rate of C, suggesting that fatigue damage evolution was effectively mitigated in the rejuvenated asphalt. The fatigue life results further confirmed this trend. ASBSMA exhibited a substantially lower *N_f_* than OSBSMA, indicating that aging impaired the stress relaxation capability and fatigue cracking resistance of the asphalt. With increasing rejuvenator dosage, the *N_f_* at both 2.5% and 5% strain levels increased progressively, demonstrating that the reactive rejuvenator effectively enhanced fatigue durability.

The improvement in fatigue performance can be attributed to the combined effects of compositional restoration and structural recovery. The waste engine oil component replenishes the light fractions depleted during aging and promotes the dispersion of heavy molecular components, thereby enhancing molecular mobility and energy dissipation during cyclic loading. Meanwhile, the epoxy functional component may facilitate interactions among aged SBS fragments and polar functional groups, contributing to partial restoration of the SBS-modified network structure. As a result, the rejuvenated asphalt exhibits improved deformation recovery capability and a reduced rate of fatigue damage propagation, leading to a longer fatigue life.

### 3.5. Comprehensive Performance Evaluation of Rejuvenated Asphalt

#### 3.5.1. Construction of the Evaluation Framework

The performance restoration of rejuvenated asphalt involves multiple aspects, including high-temperature stability, low-temperature cracking resistance, fatigue durability, and construction workability. As demonstrated in the preceding sections, individual performance indicators exhibit different responses to rejuvenator dosage. Therefore, determining the optimal dosage based on a single indicator may lead to biased or incomplete conclusions. To provide a more comprehensive assessment, a multi-criteria evaluation framework was established to quantitatively compare the overall performance of rejuvenated asphalt prepared with different rejuvenator dosages.

The evaluation system comprised four categories of performance indicators. High-temperature performance was characterized by the softening point, failure temperature, *J_nr_*, and *R*. Low-temperature performance was evaluated using the ductility at 5 °C, continuous low-temperature grading temperature (*T_c_*), and DER values at different temperatures. Fatigue performance was represented by the fatigue life (*N_f_*) at strain levels of 2.5% and 5%, while construction workability was characterized by the rotational viscosity at 135 °C. Among these indicators, the softening point, failure temperature, R, ductility, DER, and *N_f_* were classified as benefit criteria, for which larger values indicate superior performance. In contrast, *J_nr_*, *T_c_*, and rotational viscosity at 135 °C were treated as cost criteria, where lower values correspond to better performance. Construction of the evaluation framework is shown in Table 4.

#### 3.5.2. Entropy-Weight Analysis

The weights of the evaluation indicators calculated using the entropy-weight method are presented in Table 5. The entropy weight reflects the degree of variation of each indicator among different samples. Indicators with greater variability are assigned higher weights because they contribute more significantly to distinguishing the comprehensive performance of the alternatives. Among all indicators, DER at −30 °C, failure temperature, and R measured at 60 °C under 3.2 kPa exhibited the highest weights, accounting for 8.800%, 8.266%, and 8.247%, respectively. These results indicate that changes in rejuvenator dosage have a pronounced influence on low-temperature energy dissipation capability, high-temperature stability, and elastic recovery behavior.

From the perspective of indicator categories, low-temperature performance-related parameters generally received higher weights than the other indicators, suggesting that the dosage of the reactive rejuvenator plays a particularly important role in restoring the low-temperature performance of aged SBS-modified asphalt. Nevertheless, the relatively high weights assigned to failure temperature and MSCR rate indicate that excessive rejuvenator addition may adversely affect rutting resistance and elastic recovery at elevated temperatures. Therefore, optimization of rejuvenator dosage should account for the trade-off between low-temperature performance enhancement and the retention of adequate high-temperature performance.

#### 3.5.3. TOPSIS Ranking and Determination of the Recommended Dosage

In the TOPSIS method, D+ and D− represent the distances from the positive ideal solution and the negative ideal solution, respectively. A smaller D+ indicates that the evaluated alternative is closer to the optimal performance state, whereas a larger D− indicates a greater separation from the least desirable performance state. The relative closeness coefficient (C) is used to assess the overall performance of each alternative, with higher C values indicating superior comprehensive performance. The calculated results are summarized in Table 6.

As shown in Table 6, the overall performance ranking of the rejuvenated asphalts was RA-6 > RA-8 > RA-4 > RA-10 > RA-0. Among all samples, RA-6 exhibited the smallest D+ and the highest C value of 0.66504, indicating the best overall performance. In contrast, RA-0 yielded the lowest C value of 0.40166, suggesting that the addition of virgin SBS-modified asphalt alone was insufficient to fully restore the properties of the aged asphalt. The results also indicate that a higher rejuvenator dosage does not necessarily lead to better overall performance. Although RA-10 showed substantial improvements in low-temperature performance, its resistance to permanent deformation and elastic recovery capability at high temperatures were noticeably reduced, resulting in a relatively lower overall ranking. Therefore, excessive rejuvenator addition may compromise the balance among different performance attributes. Considering high-temperature stability, low-temperature cracking resistance, fatigue durability, and construction workability simultaneously, the 6% reactive rejuvenator dosage achieved the most favorable overall balance and is therefore recommended under the conditions investigated in this study.

### 3.6. Gel Permeation Chromatography

The changes in molecular-weight distribution observed by GPC are consistent with the interaction mechanism reported in our previous study. Our previous study used FTIR to investigate the interaction mechanism of the same epoxy-based reactive rejuvenator with aged SBS-modified asphalt and provided evidence of chemical interactions between the epoxy-containing component and aging-induced functional groups [41]. The present study therefore focuses on dosage-dependent performance restoration, while GPC is used as complementary evidence to characterize changes in molecular-weight distribution. It should be noted that GPC alone cannot directly identify specific chemical reactions.

**Figure 9:**
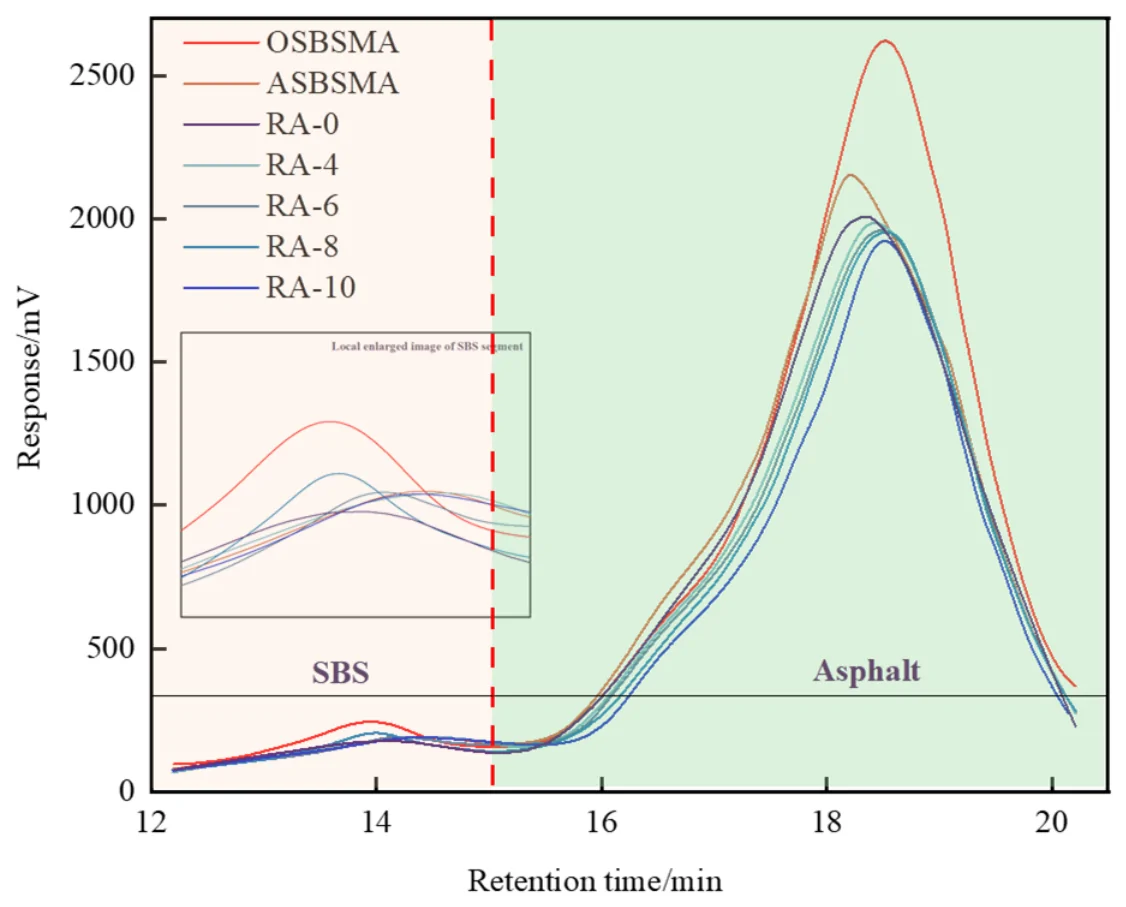
GPC results for different asphalt samples.

**Figure 10:**
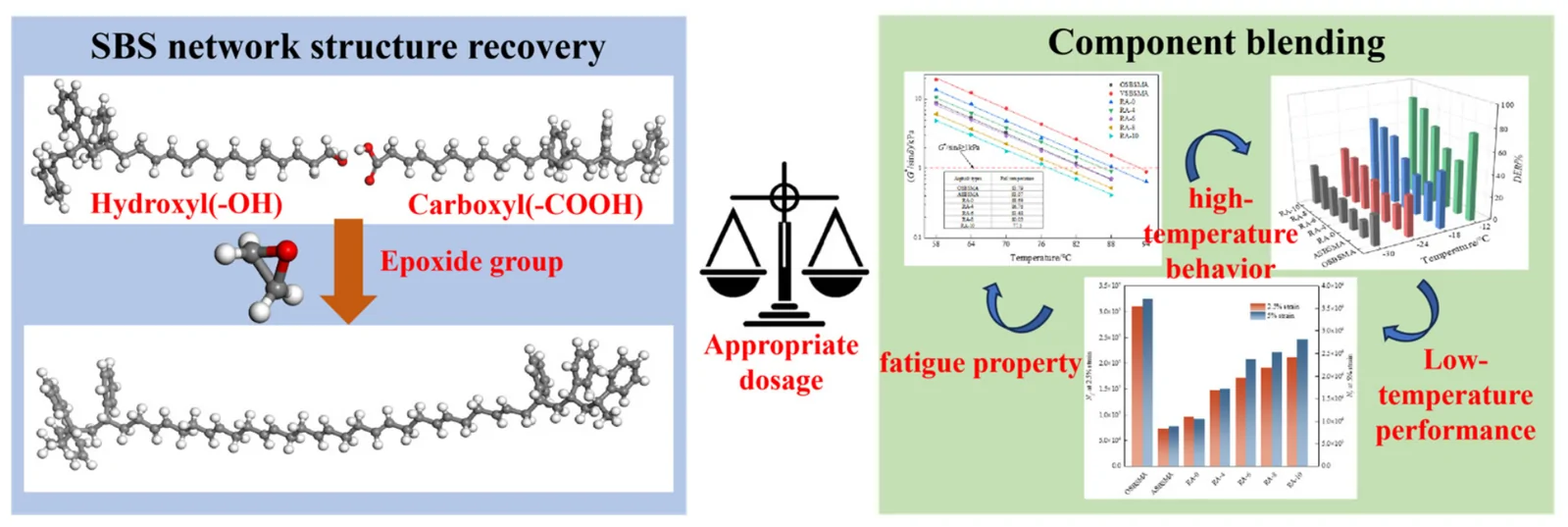
The mechanism of dosage effect.

In Figure 9, the GPC results indicate that aging caused the main peak of the asphalt phase to shift toward lower retention times, while the characteristic SBS peak shifted toward higher retention times. This phenomenon suggests that aging not only promoted the transformation of asphalt components toward higher-molecular-weight fractions but also induced degradation of the SBS molecular chains. Following the incorporation of the reactive rejuvenator, the main peak of the asphalt phase gradually shifted toward higher retention times, whereas the characteristic SBS peak progressively moved toward lower retention times. These changes indicate that the rejuvenator was capable of modifying the molecular-weight distribution of the aged asphalt and facilitating partial restoration of the degraded SBS structure. It should be noted that increasing the rejuvenator dosage does not necessarily lead to a continuous improvement in overall performance. Although increasing the dosage improves the flexibility and workability of the aged binder, an excessive addition may reduce its high-temperature deformation resistance. Therefore, the rejuvenator dosage should be determined by balancing the restoration of low-temperature and fatigue properties against the retention of adequate high-temperature performance. This interpretation is consistent with the entropy-weight TOPSIS analysis, which identified 6% as the recommended dosage among the investigated dosage levels under the conditions considered in this study. The mechanism of the dosage effect is illustrated in Figure 10.

## 4. Conclusions

This study proposed a multi-performance-based framework for determining the dosage of a reactive rejuvenator for field-aged SBS-modified asphalt under high-RAP conditions. The main conclusions are as follows:

(1) The reactive rejuvenator produced a clear performance trade-off. Increasing rejuvenator dosage improved low-temperature flexibility, stress relaxation, fatigue resistance, energy dissipation, and workability, whereas an excessive addition reduced high-temperature deformation resistance and elastic recovery. Therefore, rejuvenator dosage cannot be determined by maximizing a single performance indicator but should be selected by balancing competing performance requirements.
(2) The high- and low-temperature results quantitatively demonstrated this dosage-dependent behavior. The failure temperature decreased from 88.63 °C for RA-0 to 77.50 °C for RA-10, while RA-6 exhibited a failure temperature of 83.48 °C, close to that of OSBSMA (83.79 °C). At −18 °C, DER increased from 36.7% for RA-0 to 70.1% for RA-10, confirming the enhanced energy-dissipation and stress-relaxation capability produced by the rejuvenator. These results demonstrate that improved low-temperature performance is accompanied by progressive loss of high-temperature resistance at excessive dosages.
(3) The entropy-weight TOPSIS method effectively integrated high-temperature, low-temperature, fatigue, and workability indicators. The comprehensive ranking was RA-6 > RA-8 > RA-4 > RA-10 > RA-0, with RA-6 exhibiting the highest relative closeness coefficient of **0.66504**. The agreement between this ranking and the individual performance trends supports the feasibility of the proposed framework for multi-performance-based rejuvenator dosage determination.
(4) GPC showed systematic changes in molecular-weight distribution after rejuvenation, providing supplementary molecular-scale information for interpreting the observed performance restoration. However, these changes should not be interpreted as direct evidence of specific chemical reactions. For the material system investigated, **6% is recommended among the tested dosage levels**, and is not a universally applicable optimum.

Several limitations of this study should be acknowledged. First, the investigation was conducted primarily at the binder scale using field-aged SBS-modified asphalt obtained from a single RAP source, and only one reactive rejuvenator formulation was evaluated. Second, the fixed virgin-to-aged binder ratio used in this study may not represent all high-RAP recycling conditions. Third, aggregate–binder interactions and mixture-level performance were not considered. In addition, FTIR characterization was not conducted in the present study; therefore, the specific chemical interactions associated with the epoxy-containing component cannot be directly verified from the current experimental data. Consequently, the recommended 6% dosage should be regarded as material-dependent rather than universally applicable. Future studies should validate the proposed framework using different RAP sources, aging levels, rejuvenator formulations, mixture compositions, and field conditions, together with combined chemical and molecular characterization.

## Figures and Tables

**Table 1:** Basic properties of virgin and aged asphalt.

Property	Material		Specification Requirements
	Virgin Asphalt	Aged Asphalt	
Penetration (25 °C, 0.1 mm)	69.7	20.7	60~80
Softening point (°C)	72.5	76.4	≥55
Ductility (5 & 15 °C, mm)	35	7	≥30
Viscosity (135 °C, Pa·s)	1.230	2.435	≤3

**Table 2:** Properties of the rejuvenator.

Property		Result	Specification Requirement
Viscosity (60 °C, cSt)		172	50~175
Saturate content (%)		15	≤30
Aromatic content (%)		37	Measured
TFOT (163 °C, 5 h)	Mass variation (%)	−1.65	≥−4, ≤4
	Aromatic content (%)	1.6	<3
Flash point (°C)		238	>220

**Table 3:** Research program for different asphalt samples.

Sample	Virgin Asphalt (%)	Aged Asphalt (%)	Reactive Rejuvenator Dosage (%)	Number	Tests Performed
OSBSMA	100	0	0	3	Conventional, DSR, MSCR, BBR, LAS, GPC
ASBSMA	0	100	0		
RA-0	20	80	0		
RA-4	20	80	4		
RA-6	20	80	6		
RA-8	20	80	8		
RA-10	20	80	10		

**Table 4:** Construction of the evaluation framework.

Performance Indicators		Property
High-temperature performance	Softening point	Positive indicator
	Failure temperature	Positive indicator
	*J_nr_* at 60 °C and 3.2 kPa	Negative indicator
	*R* at 60 °C and 3.2 kPa	Positive indicator
	*J_nr_* at 70 °C and 3.2 kPa	Negative indicator
	*R* at 70 °C and 3.2 kPa	Positive indicator
Low-temperature performance	Ductility	Positive indicator
	*T_C_*	Negative indicator
	DER at −18 °C	Positive indicator
	DER at −24 °C	Positive indicator
	DER at −30 °C	Positive indicator
Fatigue performance	*N_f_* at 2.5% strain	Positive indicator
	*N_f_* at 5% strain	Positive indicator
Workability	Viscosity at135 °C	Negative indicator

**Table 5:** Information entropy and weights of performance indicators for recycled asphalt.

Performance Indicator	Information Entropy, *H*	Weight, *ω* (%)
Softening point	0.822	7.001
Failure temperature	0.79	8.266
*J_nr_* at 60 °C and 3.2 kPa	0.852	5.821
*R* at 60 °C and 3.2 kPa	0.791	8.247
*J_nr_* at 70 °C and 3.2 kPa	0.832	6.627
*R* at 70 °C and 3.2 kPa	0.827	6.800
Ductility	0.815	7.284
*T_C_*	0.830	6.696
DER at −18 °C	0.812	7.409
DER at −24 °C	0.821	7.057
DER at −30 °C	0.777	8.800
*N_f_* at 2.5% strain	0.837	6.438
*N_f_* at 5% strain	0.829	6.739
Viscosity at135 °C	0.827	6.816

**Table 6:** The calculated results.

Asphalt Type	D+	D−	C	Ranking
RA-0	0.21584	0.14489	0.40166	5
RA-4	0.14031	0.16925	0.54673	3
RA-6	0.09361	0.18586	0.66504	1
RA-8	0.11217	0.18137	0.61789	2
RA-10	0.17589	0.20331	0.53615	4

## Data Availability

The original contributions presented in this study are included in the article. Further inquiries can be directed to the corresponding author.
